# HAI Peptide and Backbone Analogs—Validation and Enhancement of Biostability and Bioactivity of BBB Shuttles

**DOI:** 10.1038/s41598-018-35938-8

**Published:** 2018-12-18

**Authors:** Pol Arranz-Gibert, Roger Prades, Bernat Guixer, Simón Guerrero, Eyleen Araya, Sonia Ciudad, Marcelo J. Kogan, Ernest Giralt, Meritxell Teixidó

**Affiliations:** 1 Institute for Research in Biomedicine (IRB Barcelona), Barcelona Institute of Science and Technology (BIST), Baldiri Reixac 10, Barcelona, E-08028 Spain; 2 Department of Pharmacological and Toxicological Chemistry, Faculty of Pharmaceutical Sciences, University of Chile, Sergio Livingstone, 1007 Independencia Santiago, Chile; 3 Advanced Center for Chronic Diseases (ACCDiS), Sergio Livingstone, 1007 Independencia Santiago, Chile; 4 Departamento de Ciencias Químicas, Facultad de Ciencias Exactas, Universidad Andres Bello, Av. Republica 275, Santiago, Chile; 5 Department of Inorganic and Organic Chemistry, University of Barcelona, Martí i Franquès 1-11, Barcelona, E-08028 Spain

## Abstract

Low effectiveness and resistance to treatments are commonplace in disorders of the central nervous system (CNS). These issues concern mainly the blood-brain barrier (BBB), which preserves homeostasis in the brain and protects this organ from toxic molecules and biohazards by regulating transport through it. BBB shuttles—short peptides able to cross the BBB—are being developed to help therapeutics to cross this barrier. BBB shuttles can be discovered by massive exploration of chemical diversity (*e.g*. computational means, phage display) or rational design (*e.g*. derivatives from a known peptide/protein able to cross). Here we present the selection of a peptide shuttle (HAI) from several candidates and the subsequent in-depth *in vitro* and *in vivo* study of this molecule. In order to explore the chemical diversity of HAI and enhance its biostability, and thereby its bioactivity, we explored two new protease-resistant versions of HAI (*i.e*. the *retro*-D-version, and a version that was *N*-methylated at the most sensitive sites to enzymatic cleavage). Our results show that, while both versions of HAI are resistant to proteases, the *retro*-D-approach preserved better transport properties.

## Introduction

From a therapeutic perspective, peptides are chemical structures that fall half-way between small molecules and biologics (*e.g*. antibodies). The latter show a better profile to interact with high affinity with the target molecule; however, their complexity does not allow their chemical synthesis and makes it difficult to obtain them as well-defined products^1^. Furthermore, such macromolecules can give rise to immunogenicity concerns and they are not suitable for oral delivery therapies. In contrast, small molecules have opposite properties. Peptides can be synthesized chemically through a well-established methodology, *i.e*. solid-phase peptide synthesis (SPPS)^2^, and can be well-characterized through analytical methods. In addition, their short length reduces the risk of triggering an immunological response, while having a sufficient active surface for high affinity and specific interaction with the target molecule^3^.

In this regard, research efforts are being channeled into peptides that have the capacity to cross the blood-brain barrier (BBB), namely BBB shuttles^4,5^, and that can thus facilitate the transport of molecules that are otherwise unable to reach the brain parenchyma alone. The BBB is an active and selective barrier that protects the central nervous system (CNS)^6–8^. Thus, BBB shuttles allow the delivery of therapeutics into the CNS through a non-invasive approach. Two clearly differentiated routes, namely passive diffusion (*i.e*. lipophilic pathway)^9^ and active transport (mainly, adsorptive- or receptor-mediated transcytosis, AMT and RMT, respectively), are the most suitable approaches for drug delivery to the brain^10^. The former is conveniently used for small cargos, while the latter enables the delivery of large therapeutics, such as proteins and nanoparticles (NPs)^10,11^.

Discovery of peptide BBB shuttles can be pursued by extensive exploration of chemical diversity, rational design from proteins that interact with cellular receptors that are internalized by endocytosis (and can be translocated by transcytosis). Here, we report (1) the study of six peptides (TAT, Antennapedia, R_8_, SAP, RVG and HAI) known to be internalized by adsorptive- or receptor-mediated endocytosis (AME and RME, respectively), (2) the subsequent deeper study *in vitro* and *in vivo* of the best hit (HAI) and (3) the improvement of its bioactivity by means of backbone redesign to enhance resistance to proteases.

## Results and Discussion

### Selection of Initial Candidates

Discovered in 1988, HIV TAT 48–57 peptide derives from the transcriptional *trans*-activator protein of the human immunodeficiency virus 1 (HIV-1)^12,13^. TAT is one of the most widely used molecular beacons for cellular drug delivery^14^, and it has already been used as a BBB shuttle^15–19^, including the transport of quantum dots (QDs)^20^, which enabled to study the mechanism underlying the capacity of this peptide to transport NPs. Antennapedia, also known as penetratin^21^, was discovered in 1994 and, together with TAT, it was one of the first cell-penetrating peptides (CPPs) to be reported. Derived from the third helix of the Antennapedia homeodomain of *Drosophila*, it has been used as a vector for the delivery of range of cargos into cells^22^, for intestinal absorption^23^, and for brain delivery^24^. Inspired by the common features of TAT and Antennapedia, Futaki *et al*. designed and synthesized a library of oligoarginine peptides (R_n_)^25^.These peptides translocate across cell membranes in a similar manner to that used by TAT and Antennapedia, especially the R_8_ and R_12_ peptides, which show high penetration efficiency. Although there are some concerns about their cytotoxicity^26^, oligoarginine peptides are among the most efficient CPPs with respect to translocation across cell membranes. Their use as BBB shuttles has not been reported to date. Sweet Arrow Peptide (SAP) has a Pro-rich structure and is derived from the *N*-terminal domain of γ-zein, a maize storage protein^27^. One of its main features is its efficiency to translocate across cell membranes without presenting toxicity, both *in vitro*^27^ and *in vivo*^28^. SAP was discovered by our group, and to date there are no published data about its capacity as a BBB shuttle. A 29-amino acid peptide derived from rabies virus glycoprotein, so-called RVG, targets the nicotinic acetylcholine receptor (nAChR)^29^, widely expressed in the brain, including the endothelial cells of brain capillaries^30^. This peptide has been successfully used as a BBB shuttle for the delivery of small interfering RNA (siRNA) in mice^31^. Finally, the peptide HAI was found by Lee *et al*. by phage display against the human transferrin receptor (TfR)^32^. Highly expressed in brain capillaries, TfR mediates the delivery of iron to the brain^33^. It is also expressed in choroid plexus epithelial cells and neurons^34^. One of the main advantages of this peptide is that it interacts in a region of the TfR that does not overlap with the native binding site of transferrin, thereby avoiding physiological effects on the protein function *in vivo* and consequently making this peptide very attractive from the therapeutic point of view. HAI has been studied for diverse applications such as tumor-targeting^35^ and oral drug delivery^36^. Its potential as a BBB shuttle has recently been addressed by Kuang *et al*.^37^. Given that other peptides interacting with the TfR have the capacity to cross the BBB^10^ and that the BBB endothelium is characterized by high presence of TfR^38^—a feature that can selectively enhance brain targeting—here we expand on the potential applications of HAI as a BBB shuttle.

### Internalization and Transport of Peptide Candidates —*in vitro* Studies

Screening of compounds as potential candidates for specific therapeutic applications usually involves *in vitro* experiments. Their implementation helps to both simplify the system of study and reduce the experimental cost and the number of experimentation animals used. In recent decades, such models have been extended to the study of the BBB^39,40^, thus enabling the quantification of transport for individual compounds. Here we initially screened the selected BBB shuttle candidates in a series of *in vitro* BBB models in order to select the peptide with the best performance for further study and development.

**Figure 1:**
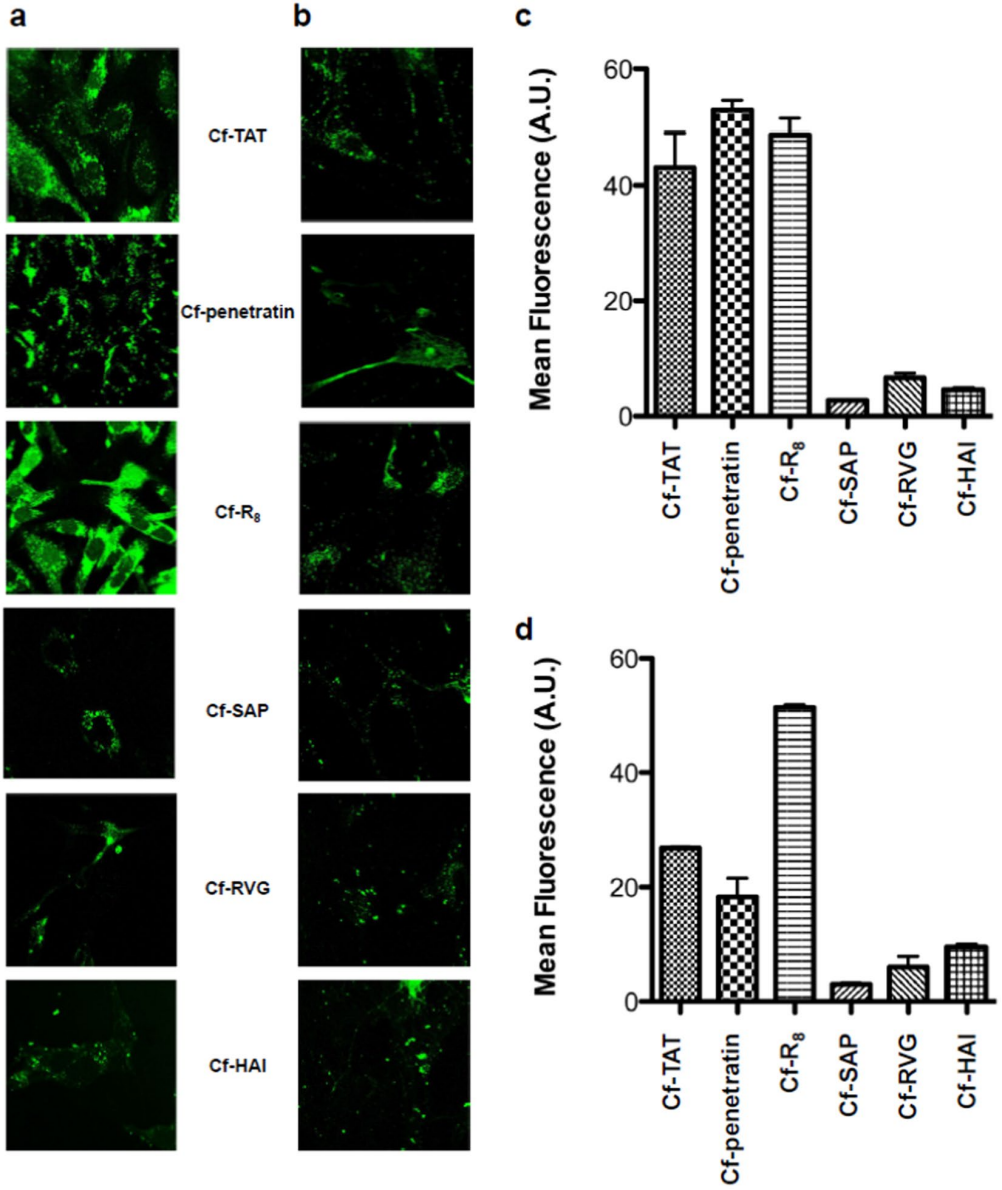
Peptide internalization by cells. CLSM images of (**a**) BBECs and (**b**) rat astrocytes incubated with the candidates at a concentration of 50 μM (R_8_ was incubated at a concentration of 15 μM) at 37 °C for 3 h. Flow cytometry results after incubating (**c**) BBECs and (**d**) rat astrocytes with each peptide under the same experimental conditions as previously described. Data are expressed as the mean ± SD.

Initially, peptides labeled at the *N-*terminus with carboxyfluorescein (Cf) were incubated with bovine brain endothelial cells (BBECs) or rat astrocytes at 50 μM (except R_8_, assayed at 15 μM to avoid cytotoxicity) in order to determine their internalization capacity, a necessary but not sufficient condition to cross the BBB. Cells were then analyzed by confocal laser scanning microscopy (CLSM) and by flow cytometry (Fig. 1). The data from these experiments revealed that the three CPPs (TAT, penetratin and R_8_) showed the highest internalization profiles in both cell types. Nevertheless, greater internalization does not necessarily equate to better transcytosis, since high retention in cells would prevent the peptide from crossing from the apical to the basal side (brain parenchyma).

In a subsequent step we evaluated the transport capacity of these peptides using two *in vitro* barrier models, namely in an intestine (Caco-2 assay)^41^ and a BBB model^39,40^ (Table 1). In these models, two compartments are separated by a membrane containing a monolayer of endothelial cells which mimics either the intestinal barrier or the BBB. One compartment contains the peptide, which is incubated for 2 h. The amount of peptide in each compartment is then analyzed to determine apparent permeability (*P*_*app*_, in cm/s) and transport (*T*, in %). The integrity of the compound in the acceptor compartment is assessed by mass spectrometry (MS). Our results show that only penetratin and HAI crossed the intestinal barrier (and only penetratin, R_8_ and HAI were detected by MALDI-TOF MS in acceptor wells) while HAI showed the best performance in the BBB model (only TAT was not detected by MALDI-TOF MS in acceptor wells). Thus, we turned our attention to HAI to further study its potential as BBB shuttle.

**Table 1:** Caco-2 and *in vitro* bovine BBB model permeability results (mean ± SD).

Peptide	Caco-2		BBB	
	*P*_*app*_ (×10^−10^ cm/s)	*T* (%)	*P*_*app*_ (×10^−6^ cm/s)	*T* (%)
TAT	0	0	0	0
Penetratin	4.2 ± 1.3	1.6 ± 0.5	3.0 ± 0.6	1.8 ± 0.2
R_8_	0	0	0.60 ± 0.06	0.68 ± 0.07
SAP	0	0	5.5 ± 1.5	5 ± 3
RVG	0	0	0.28 ± 0.02	0.29 ± 0.03
HAI	2.0 ± 0.8	0.7 ± 0.3	18 ± 2	16 ± 3

Candidates were incubated for 2 h at 37 °C using HBSS as buffer and then analyzed by RP-HPLC to determine transport and *P*_*app*_.

### Studying the Selected Candidate—Mechanistic *in vitro* Studies, *in vivo* Transport Efficiency and Biostability

**Figure 2:**
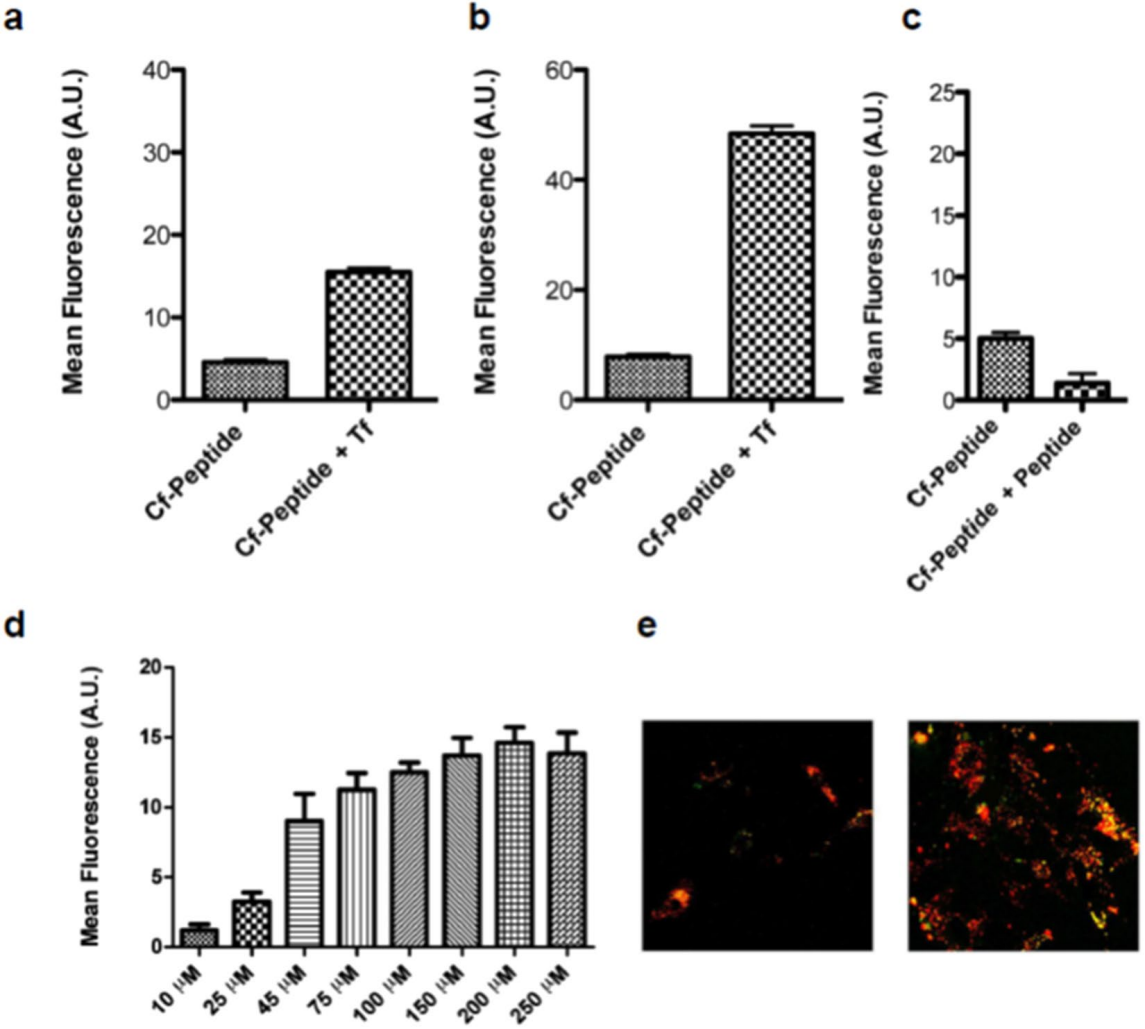
Studies of the internalization mechanism of HAI: flow cytometry results after incubating (**a**) bovine brain endothelial cells (BBECs) or (**b**) rat astrocytes with Cf-HAI at 50 μM in the absence or presence of transferrin, (**c**) co-incubation of Cf-HAI with HAI (competition assay), (**d**) incubation of BBECs with a range of concentrations of Cf-HAI, and (**e**) co-incubation of (left) BBECs or (right) rat astrocytes (co-localization experiments) with Cf-HAI at 50 μM with fluorescently labeled transferrin (AlexaFluor555). Data are expressed as mean ± SD.

We devised a series of experiments to ensure that HAI delivery is TfR-dependent, and at the same time to establish whether it competes with Tf—an observation previously reported^32,42^. HAI transport (cellular internalization) was promoted by the addition of Tf, the natural ligand of TfR, which might induce the internalization and transcytosis of the aforementioned receptor by the cells (Fig. 2a,b). Thus, the peptide did not compete with Tf for the same binding pocket at TfR. Moreover, competition assays revealed that HAI competes with itself for internalization (Fig. 2c), and incubation of cells with increasing concentrations of the peptide led to the saturation of internalization (Fig. 2d). Both observations indicate that HAI is actively transported. Finally, we show that this peptide co-localizes with Tf when cells are incubated with carboxyfluorescein (Cf)-labeled HAI (Cf-HAI) and Alexa555-Tf (Fig. 2e). This observation thus demonstrates that the internalization of HAI occurs through clathrin-mediated endocytosis, as already described for the TfR-Tf pair.

Furthermore, HAI was characterized by circular dichroism (CD). This approach revealed a profile like that of a random coil conformation (Figure S2), with a negative band at 197 nm and a weak band at 220 nm. Toxicity assays demonstrated that the peptide is not toxic for BBECs or rat astrocytes, when these cells are incubated with the peptide at a concentration of 50 μM for 24 h (see Figure S3a,b).

**Figure 3:**
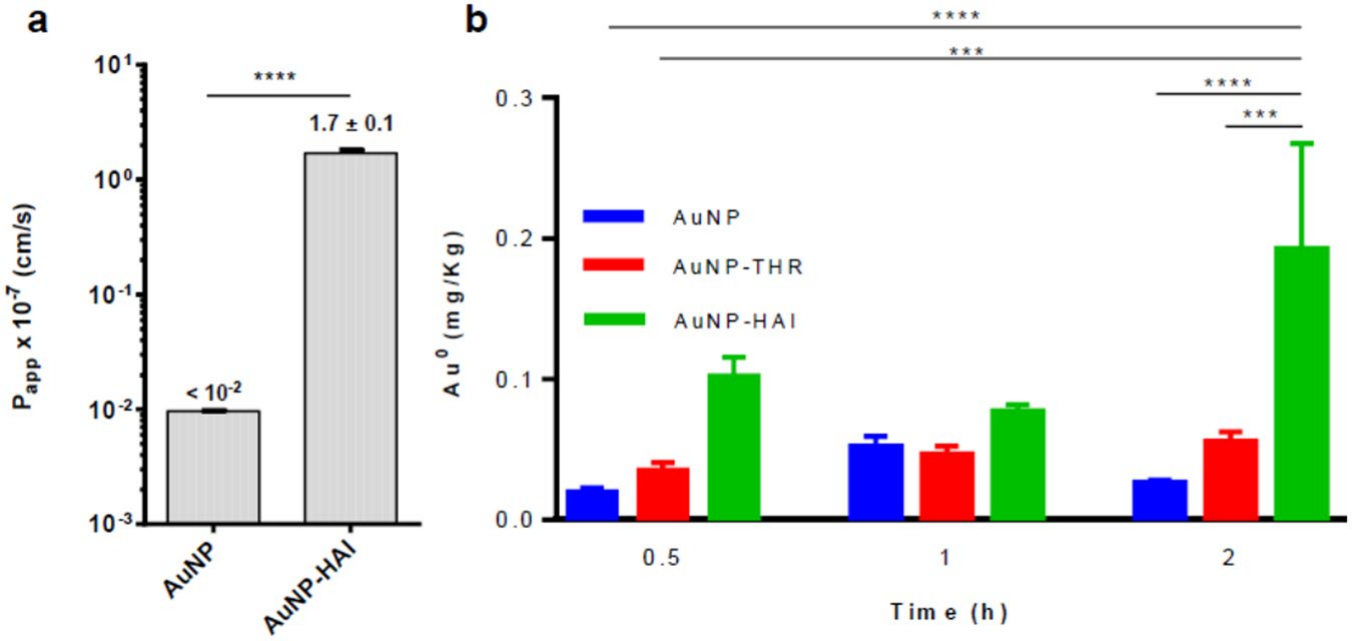
*In vitro* and *in vivo* transport of AuNPs (mean ± SD). Using the same *in vitro* bovine BBB transport model used for the peptides analyzed previously; (**a**) apparent permeability (*P*_*app*_) was obtained for AuNPs and AuNP-HAI. Then, (**b**) *in vivo* studies were performed in rats to confirm the capacity of HAI to deliver AuNPs into the CNS. In these experiments, the corresponding AuNPs were injected *i.p*. using 1.86 mg gold content per kg of body weight. The gold content in the brain was determined by INAA at three timepoints (0.5, 1, 2 h). In (**a**) data was analyzed using the t-test, ****P < 0.0001. In (**b**), the one-way ANOVA analysis was applied, *P < 0.5, **P < 0.01, ***P < 0.001, ****P < 0.0001. Data for AuNP-THR obtained from Prades *et al*.^11^.

To study the *in vitro* and *in vivo* potential of HAI to deliver a larger cargo to the CNS, we decorated gold nanoparticles (AuNPs) with this peptide (see full characterization in Figures S4–S6, Table S2). We first discarded cytotoxicity arisen (Figure S7) by these constructs to subsequently evaluate their transport in the previously used bovine BBB *in vitro* model (Fig. 3a)^39,40^. We ensured that the size of our AuNPs and conjugates were adequate (*i.e*. small enough) for *in vivo* purposes but at the same time larger than 20 nm and thus avoiding alteration of the peptide structure (Figure S6)^43^. Incubation of the cells with AuNP-HAI at a concentration of 5 nM caused an increase in transport by more than two orders of magnitude, up to 1.7 (0.1) × 10^−7^ cm/s, compared to AuNPs (0.97 (0.003) × 10^−9^ cm/s). In addition, we compared these results with those of a previously studied BBB shuttle (THR) carrying the same NPs Prades *et al*. HAI conjugates showed slightly higher permeability than AuNP-THR, 1.40 (0.07) × 10^−7^ cm/s^11^.

To compare the delivery of AuNP, AuNP-HAI and AuNP-THR to the brain, *in vivo* experiments in rats were performed (the latter results, compared from Prades *et al*.)^11^. Animals were euthanized at different times (0.5, 1 and 2 h) after *i.p*. injection. Brains were excised after PBS perfusion. They were lyophilized and dried to proceed with their gold-content quantification by instrumental neutron activation analysis (INAA)—samples are irradiated with neutrons, converting ^197^Au to ^198^Au and leading to γ ray emission that can be quantified. Trace levels of gold found in the CNS are around 2 × 10^5^ mg/kg fresh tissue^44^. Our samples showed a significantly much higher gold content. Furthermore, the conjugation of AuNPs to HAI and THR increased the concentration of this metal in the brain with respect to naked AuNPs (Fig. 3b). Remarkably, AuNP-HAI showed greater penetration than AuNP-THR.

**Figure 4:**
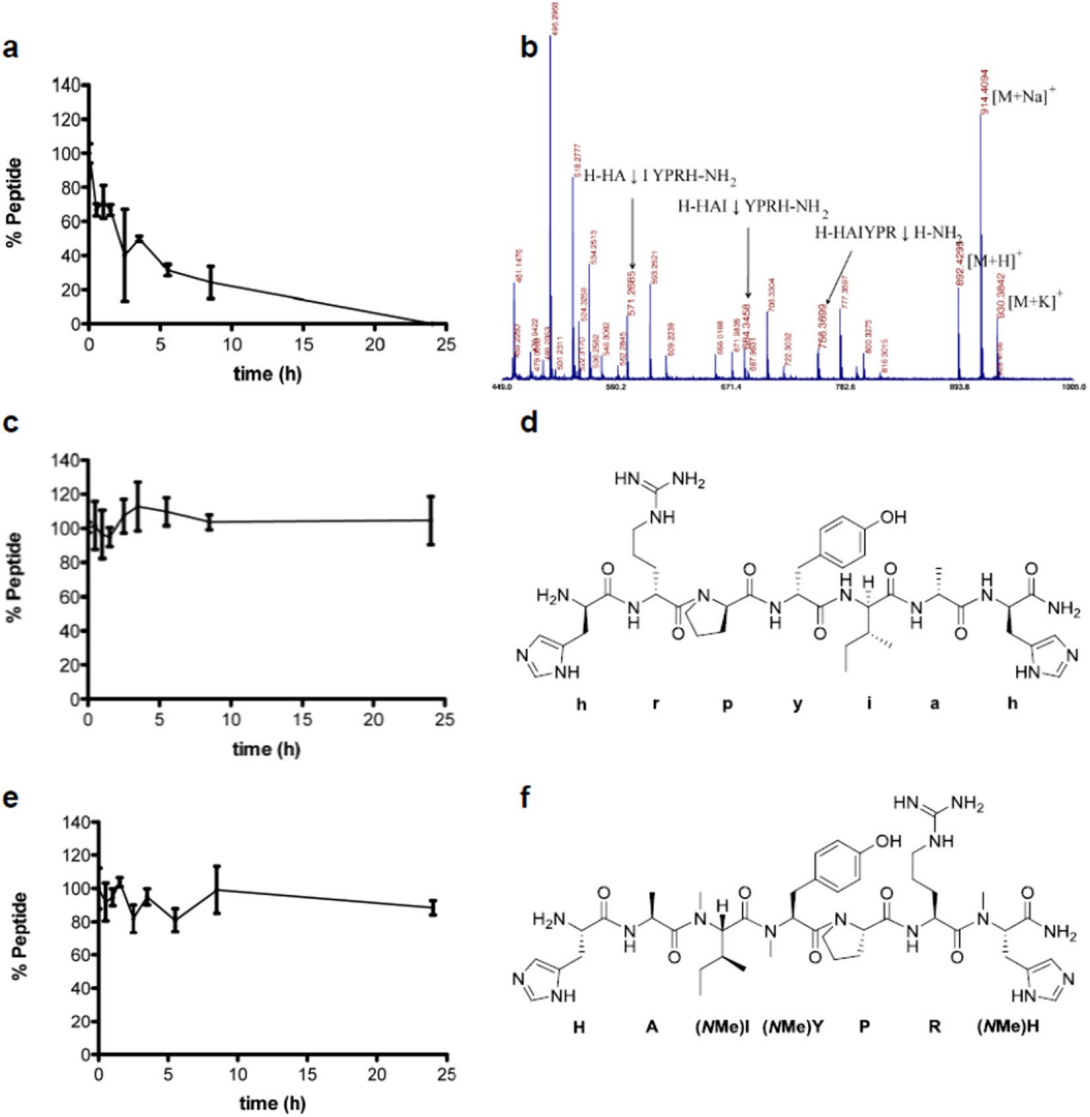
Stability of HAI, *r*D-HAI and (*N*Me)HAI in human serum: (**a**,**c**,**e**) graphic representation of the remaining peptide *vs*. time for each peptide (mean ± SD), respectively; (**b**) MALDI-TOF traces of HAI peptide after 30 min; (**d**,**f**) structures of the peptides *r*D-HAI and (*N*Me)HAI, respectively.

HAI showed excellent transport through the BBB, thus emerging as a promising BBB shuttle. Nevertheless, peptides formed by L-amino acids have a short *in vivo* half-life in serum, which can reduce their potential bioactivity. The sensitivity of this peptide to proteases was tested at a concentration of 150 μM in HBSS/human serum 90:10 (v/v) for 24 h. At several time points, an aliquot was extracted, precipitated serum proteins with MeOH and then analyzed by RP-HPLC. The peptide showed a half-life in serum of around 5 min, a much shorter time than that expected for a drug-like compound. The potential proteolytic sites were analyzed by MALDI-TOF, which revealed three main proteolytic sensitive sites: H-HA↓I↓YPR↓H-NH_2_ (where ↓ shows the cleavage between two consecutive residues; see Figs 4a,b and S9,S10).

### Tuning Protease-Resistance: *N*-Methylation of Labile Positions *vs*. the *Retro*-D-Version Approach

Several strategies can be used to increase the *in vivo* stability of a peptide composed of L-amino acids. Here we selected the *N*-methylation of sites sensitive to proteolysis and the *retro*-enantio approach, which is a topological mimic with a reversed sequence order and inversed stereochemistry in each α-carbon (*i.e*. made by D-amino acids)^45^. The *N-*methylated peptide designed was H-HA(*N*Me)I(*N*Me)YPR(*N*Me)H-NH_2_, which contained the sensitive sites (see previous section) protected with an *N-*methyl group^46,47^, namely (*N*Me)HAI. The *retro-*enantio peptide (or *retro*-D) was H-hrpyiah-NH_2_ (where the lowercase letter denotes D-amino acid), namely *r*D-HAI. Thus, we synthesized and studied the stability of these two peptides in serum (Figs 4c–f and S9). Both displayed excellent protease resistance (half-life above 24 h).

Characterization of peptides by CD revealed random coil conformation like that of the parent version (Figure S2), meaning that in solution they do not adopt a preferential conformation. Similarly, like the parent peptide, the two protease-resistant analogs did not show toxicity up to 50 μM, as indicated by the MTT assay at 24 h (Figure S3c,d).

**Figure 5:**
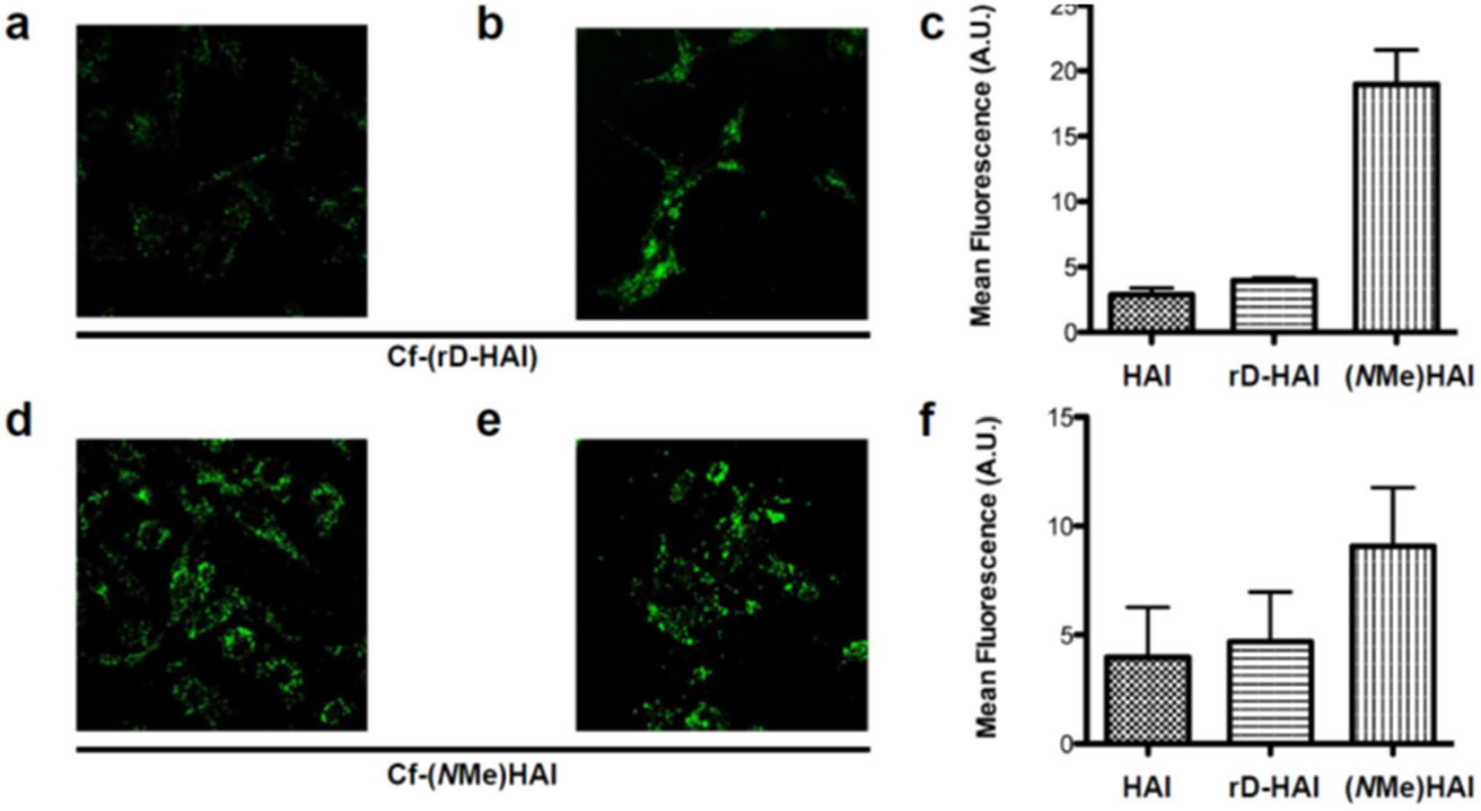
Peptide internalization by cells. Confocal laser scanning microscopy (CLSM) images of (**a**,**d**) BBECs and (**b**,**e**) rat astrocytes incubated with the candidates at a concentration of 50 μM at 37 °C for 3 h. Flow cytometry results (mean ± SD) after incubating (**c**) BBECs and (**f**) rat astrocytes with the candidates at a concentration of 50 μM (peptide 3 was incubated at 15 μM) for 3 h at 37 °C.

We then studied the capacity of these peptides to be internalized by and transported through the endothelium of the BBB or intestine. Thus, we evaluated their transport capacity using the two previously used *in vitro* barrier models, *i.e*. Caco-2 and BBB assays. All the peptides were detected by MALDI-TOF MS in the acceptor wells. The (*N*Me)HAI analog displayed a two-fold increase in internalization with respect to the parent version (Fig. 5), while *r*D-HAI maintained the internalization rate in BBECs and rat astrocytes.

The *in vitro* barrier model of the BBB revealed discrepancies in transport rate (Table 2). *r*D-HAI displayed the highest value (around 1.5 times the rate of the parent version), while (*N*Me)HAI recorded an intermediate value. The discrepancy between internalization and transport of (*N*Me)HAI is probably due to an increase in lipophilicity after methylation of the backbone, thereby enhancing internalization by passive diffusion but not favoring the transcytosis. To ensure that *r*D-HAI crosses the BBB through active transport, we proceeded to test its mechanism of transport using a human model of the BBB. The peptide was assayed at increasing concentrations in an *in vitro* human BBB model (Figure S12), observing saturation of transport—typical of active transport.

**Table 2:** Caco-2 and *in vitro* bovine BBB model permeability results (mean ± SD).

Peptide	Caco-2		BBB	
	*P*_*app*_ (×10^−10^ cm/s)	Peptide	*P*_*app*_ (×10^−6^ cm/s)	*T* (%)
HAI	2.0 ± 0.8	0.7 ± 0.3	9 ± 5	10 ± 3
*r*D-HAI	1.2 ± 1.0	0.4 ± 0.3	14 ± 2	17 ± 3
(*N*Me)HAI	0.60 ± 0.01	0.200 ± 0.003	11.7 ± 1.3	13.9 ± 1.6

Candidates were incubated for 2 h at 37 °C using HBSS as buffer, and then analyzed by RP-HPLC to determine transport and *P*_*app*_.

## Conclusion

We first selected the peptide HAI among a set of peptides known to be endocytosed by AME or RME, and then further explored its capacity as a shuttle. The peptide was able to deliver AuNPs to the brains of rats and showed better transport capacity than a previous peptide studied by our laboratory (namely THR). In addition, two strategies through which to increase the protease-resistance of a peptide, namely the use of the *retro*-D-version or the *N*-methylation of the most sensitive sites, were applied to HAI. While (*N*Me)HAI showed an enhancement in internalization, its permeability through *in vitro* transport models did not increase—the hydrophobicity increase by *N*-methylation of the backbone may have enhanced internalization by other mechanism, *i.e*. passive diffusion. In contrast, the *retro*-D-approach appeared to preserve the shuttle properties (similar internalization), while enhancing its biostability and permeability; obtaining a lead BBB shuttle from a validated hit (HAI) using *in vitro* and *in vivo* experiments.

## Methods

### Materials and Methods

Protected amino acids and resins were supplied by Luxembourg Industries (Tel-Aviv, Israel), Neosystem (Strasbourg, France), Calbiochem-Novabiochem AG (Laüfelfingen, Switzerland), PolyPeptide Laboratories (Torrance, CA, USA), Bachem AG (Bubendorf, Switzerland), and Iris Biotech (Marktredwitz, Germany). DIEA and ninhydrin were from Fluka Chemika (Buchs, Switzerland). Solvents for peptide synthesis and RP-HPLC were supplied by Scharlau or SDS (Barcelona, Spain). Trifluoroacetic acid was purchased from KaliChemie (Bad Wimpfen, Germany). The other chemicals used were from Aldrich (St. Louis, MO, USA) and were of the highest purity commercially available. Mass spectra were recorded on an Applied Biosystems 4700 MALDI-TOF spectrometer (PE Applied Biosystems, Foster City, CA, USA), using an ACH matrix. High-resolution mass spectra were recorded on a Synapt HDMS (Waters, Manchester, UK) and on a LTQ-FT Ultra (Thermo Scientific, Waltham, MA, USA). RP-HPLC chromatograms were recorded on a Waters model Alliance 2695 with photodiode array (PDA) detector 996 from Waters (Waters, Milford, CT, USA) using a SunFire C_18_ column (150 × 4.6 mm × 5 µm, 100 Å, Waters); solvents: H_2_O (0.045% TFA) and CH_3_CN (0.036% TFA); flow rate of 1 mL/min; and software Millenium version 4.0. HPLC-MS [Waters model Alliance 2796, quaternary pump, Waters 2487 with UV/Vis dual absorbance detector, ESI-MS model Micromass ZQ and Masslynx version 4.0 software (Waters)] was done using a SunFire C_18_ column (150 × 3.9 mm × 5 µm, 300 Å, Waters); solvents: H_2_O (0.1% formic acid) and CH_3_CN (0.07% formic acid); and flow rate of 1 mL/min. The products were purified in a 2545 binary gradient module, a 2767 manager collector, and a 2998 photodiode array (PDA) detector (Waters), with Masslynx version 4.1 (Waters), and a SunFire C_18_ column (150 × 10 mm × 5 µm, 100 Å, Waters); solvents: H_2_O (0.1% TFA) and CH_3_CN (0.1% TFA); and flow rate of 3 mL/min. Graphics were performed using GraphPad Prism version 6.01 for Windows (GraphPad Software, La Jolla, CA, USA).

### General Protocol for SPPS

All peptides were synthesized by standard 9-fluorenylmethoxycarbonyl/*tert*-butyl (Fmoc/*t*Bu) solid-phase peptide synthesis (SPPS). Syntheses were performed on a 200-µmol scale using a Fmoc-Rink-Amide *p*-MBHA resin^2,48^. Standard solid-phase peptide elongation and other solid-phase manipulations were done manually in polypropylene syringes, each fitted with a polyethylene porous disk at the bottom. Solvents and soluble reagents were removed by suction. Between couplings and deprotections, the resin was washed with DMF (5 × 1 min), DCM (5 × 1 min), and DMF (5 × 1 min), using 5 mL of solvent/g of resin each time. During couplings, the syringe was left under automatic stirring. Intermittent manual stirring was applied during deprotections. After each reaction, the Kaiser test^49^ was used to identify primary amines on the *N*-terminus of the elongating peptide on the solid support. The chloranil test^50^ was used to identify secondary amines. Resin was conditioned by washing with DCM (5 × 1 min) and DMF (5 × 1 min), followed by 20% piperidine in DMF (1 × 1 min, 2 × 10 min) to remove the Fmoc group. Finally, the resin was washed with DMF (5 × 1 min). Fmoc group was removed by treating the resin with 20% piperidine in DMF (3–4 mL/g of resin; 1 × 1 min, 2 × 10 min). For secondary amine Fmoc deprotection, resin was additionally treated with DBU, toluene, and piperidine in DMF (5:5:20:70, v/v) (1 × 10 min). *N*α-Fmoc-protected amino acids (4 eq.), TBTU (4 eq.) and DIEA (8 eq.) were used for the coupling onto primary amines. For the coupling onto proline, TBTU was replaced by PyBOP (4 eq.) and HOBt (12 eq.). Carboxyfluorescein (Cf) was coupled at the *N*-terminal part of the peptides when required using the same coupling steps.

### *N*-Methylation

Amino acid derivatives were *N*-methylated using the method described by Miller *et al*.^47^. This process can be divided into three steps: protection and activation with *o*-nitrobenzensulfonyl chloride (*o*-NBS), deprotonation and methylation, and *o*-NBS removal. This method is not suitable for the *N*-alkylation of the first amino acid anchored onto the resin. Hence, the commercially available Fmoc-(*N*α-Me)-L-His(Trt)-OH was employed as first coupled amino acid for the synthesis of H-HA(*N*Me)I(*N*Me)YPR(*N*Me)H-NH_2_ and Cf-HA(*N*Me)I(*N*Me)YPR(*N*Me)H-NH_2_. To perform the protection, *o*-NBS (3 eq.) and collidine (5 eq.) in DCM were added to the resin and left for 60 min. This step was repeated once and the completion of the reaction was monitored by the ninhydrin test. Methyl *p*-nitrobenzensulfonate (4 eq.) and 7-methyl-1,5,7-triazabicyclo[4.4.0]dec-5-ene (3 eq.) in DMF were added to the resin and left for 30 min. This step was repeated once. To remove *o*-NBS, β-mercaptoethanol (10 eq.) and DBU (5 eq.) in DMF were added to the resin and left to react for 10 min under N_2_ atmosphere. This operation was repeated once for 40 min.

### Peptide Cleavage

Final amide peptides were cleaved from the resin by treatment with a mixture of TFA/H_2_O/EDT/TIS, 94:2.5:2.5:1 (v/v) for 1 h.

### Work-Up

After evaporation of the cleavage cocktail, 20 mL of methyl *tert*-butyl ether (MTBE) was added to the plastic tube containing the dry residue. This tube was centrifuged at 2,000 × *g* for 10 min. The solvent was then discarded by decantation. This process was repeated two more times. After the last washing with MTBE, the final residue was dissolved in H_2_O/CH_3_CN (1:1) and then lyophilized.

### Peptide Purification and Characterization

Peptides were purified by RP-HPLC using a SunFire C_18_ column. Compound identity was confirmed using MALDI-TOF MS. Peptide purity was checked by RP-HPLC using a SunFire C_18_ column. Circular dichroism spectra were recorded with a Jasco 810 UV-Vis spectropolarimeter, with a Peltier CDF 426 S/426 L. The spectra were obtained in a wavelength range of 190 to 250 nm, with a time response of 4 s, a scan speed of 10 nm/min, a step resolution of 0.1 nm, and three accumulations.

### Internalization of Peptides and Confocal Laser Scanning Microscopy Analysis

Primary cultures (rat astrocytes) were seeded onto Petri dishes or glass-bottom Petri dishes at a concentration of 40·10^3^ cells/cm^2^; experiments were carried out 48 h later. On the other hand, immortalized cell lines (BBECs) were seeded onto Petri dishes or glass-bottom Petri dishes at a concentration of 21.4·10^3^ cells/cm^2^; experiments were carried out 24 h later. Then, cells were incubated with peptides at a concentration of 50 μM (except R_8_ –incubated at a concentration of 15 μM) for 3 h at 37 °C under 5% CO_2_ in DMEM. They were then washed five times with DMEM without phenol red and confocal laser scanning microscopy analysis was performed. For co-localization experiments with transferrin protein, peptides were co-incubated with AlexaFluor555-transferrin at a concentration of 50 mg/mL. To prevent crosstalk, emission signals were recorded sequentially. Confocal microscopy images were recorded using an Olympus Fluoview 500 inverted confocal microscope with a 60×/1.4 NA objective, equipped with a 488 nm line of an argon laser, and a 543 nm HeNe laser; or a Leica TCS SPE system (DM 2500) spectral confocal microscope with a 63×/1.3 and a 40×/1.15 oil CS objective, equipped with the following diode lasers: 405, 488, 532 and 635 nm.

### MTT Toxicity Assay

The assay was performed in 96-well plates, adding 3,150 cells to each well in 100 μL (immortalized cell line –*i.e*. BBECs) or 7,000 cells in 100 μL (primary cultures –*i.e*. rat astrocytes), seeded 24 h before performing the experiment. Cells were incubated with each peptide at a range of concentrations (20, 50 and 500 μM) in cellular medium for 24 h at 37 °C and 5% CO_2_. After 22 h, MTT (3-(4,5-dimethylthiazol-2-yl)−2,5-diphenyltetrazolium bromide) was added to a final concentration of 0.5 mg/mL. The cells with peptides and MTT were incubated for further 2 h. The medium was then discarded and 200 μL of 2-propanol was added to each well to dissolve the purple formazan, product of the conversion of MTT by the mitochondrial enzyme NADH dehydrogenase. The plate was kept in an orbital shaker for 30 min (15 rpm) to ensure the complete dissolution of formazan. Finally, absorbance was measured at 570 nm and cell viability percentages were calculated dividing the absorbance value of cells treated with a given peptide by the absorbance value of untreated cells. Experiments for each peptide were carried out by triplicate. Cells incubated with 1% SDS were used as control of 0% viability (value subtracted to all samples before viability calculus per each peptide).

### Synthesis and Characterization of the AuNP-Peptide

AuNPs synthesis, conjugation and characterization were performed as previously reported by Prades *et al*.^11^. Briefly, a Cys residue was introduced in the *C*-terminal end of the peptides to enable the conjugation to AuNPs. AuNPs were conjugated to the peptides by mixing a solution of AuNPs (5 nM, 10 mL) and the corresponding peptide (0,1 mg/mL, 1 mL). The free peptide was eliminated by dialysis using a MWCO 6–8000 (Spectra/Por) membrane. The peptide load of AuNPs was calculated by dividing peptide grafted to NPs and number of NPs, which was estimated according to Liu *et al*.^51^. The concentration of AuNPs in the solution was obtained from the gold-content determined by ICP-MS and the average size obtained by TEM. The peptide concentration was determined by amino acid analysis (AAA). Zeta-potential and DLS measurements were obtained in a Malvern Zetasizer Nano ZS (Malvern Instruments), at 25 °C using a 3-mm light path cuvette. The refractive index was considered 1.4. Cell viability was assessed using a tetrazolium salt reduction assay (CellTiter 96) using SH-SY5Y.

### *In vivo* Experiments with the AuNP-Peptide

All animal experimental protocols were approved by the Local Bioethics Committee of the School of Pharmacy of the University of Chile (CBE-2012-21). All methods were carried out in accordance with guidelines and regulations animal procedures of Springer Nature. Experiments were performed as previously reported by Prades *et al*.^11^. Briefly, male Sprague Dawley rats (180–200 g body weight) were randomly divided into groups of 4 animals each and one control group (treated with sodium citrate 2.2 mM). AuNPs, AuNP-HAI and AuNP-THR were injected intraperitoneally (1.86 mg gold/kg of body weight) and gold content was analyzed at three time points (0.5, 1 and 2 h). AuNP-THR conjugates data were extracted from Prades *et al*.^11^. Instrumental neutron activation analysis (INAA) was used to measure the amount of gold in brains.

### *In vitro* Bovine BBB Cell-Based Model Assay

This assay was an adapted model^10^ of the method previously published by Gaillard and de Boer^52^. Briefly, TEER was measured in all transwells (TEER > 100 Ω·cm^2^), before performing the assay. Peptides were prepared at a concentration of 200 μM in Ringer-HEPES buffer containing 20 μM Lucifer yellow (LY) lithium salt (Sigma-Aldrich) as control (P_app_ < 17·10^−6^ cm/s). The apical compartment was filled with 200 μL of the solution containing the peptide, and 800 µL of Ringer-HEPES was poured into the basal well. Three replicates of each peptide were assayed. The plate was left for 2 h in the incubator at 37 °C. Finally, the samples were collected and analyzed or frozen until analysis. LY fluorescence was measured in a 96-well plate with a Fluoroskan Ascent Microplate Fluorometer (Thermo Fisher Scientific). TfR expression was confirmed by immunohistochemistry, as subsequently described. Usually on day 8 of co-culture (maximum TEER data obtained), the cells attached to the transwell filter were rinsed with PBS and fixed in a 4% paraformaldehyde solution in PBS for 10 min at room temperature. Cells were rinsed three times with PBS, and the reactivity of aldehyde groups was blocked by addition of 50 mM NH_4_Cl in PBS for 20 min at room temperature, after which cells were washed with PBS. The next step involved the blocking of further unspecific binding and cell membrane permeabilization, which was achieved by adding a solution of 0.1% saponin and 1% BSA in PBS for 10 min at room temperature. Insert filters were then washed by immersion in PBS at room temperature. Filters were then cut using a scalpel and the filter face where cells were attached was incubated with 12 µL of the corresponding primary anti-TfR antibody in a solution of PBS and 1% BSA for 1 h. Filters were washed by immersion in PBS (2 × 5 min) at room temperature and incubated with 12 µL of the secondary antibody in a solution of PBS, 1% BSA and DAPI for 1 h. Filters were washed 4 times with PBS at room temperature and placed on a glass slide. 15 µL of Mowiol was added to each filter, and a cover slide was placed over each one. Glass slides with filters were stored at 4 °C protected from light until confocal microscopy analysis.

### Caco-2 Intestinal Barrier Model Assay

The ability of peptides to overcome the intestinal barrier was tested using Caco-2 cells. Transport studies were conducted by the BioFarma Research group at University of Santiago de Compostela. Briefly, experiments were done with cells (passage between 60 and 80) seeded on 4.67 cm^2^ filters at a density of 5.25·10^4^ cell/cm^2^. Peptides dissolved in HBSS were incubated for 2 h at a 50 μM concentration with cell layers at 37 °C and 5% CO_2_. After the experiment, the content of each compartment was directly analyzed by HPLC and then transport (in %) and *P*_*app*_ were determined.

### *In vitro* Human BBB Cell-Based Model Assay

This assay was performed using the model published by Cecchelli and co-workers^53^. Endothelial cells and pericytes were defrosted in gelatin-coated Petri dishes (Corning). Pericytes and endothelial cells were cultured in DMEM pH 6.8 or in supplemented endothelial cell growth medium (Sciencells), respectively. After 48 h, pericytes (50,000 cells/well) and endothelial cells (80,000 cells/well) were seeded in gelatin-coated 12-well plates or in Matrigel-coated 12-well Transwell inserts (Corning), respectively. Medium was changed every 2–3 days and assays were performed 7–8 days after seeding. Lucifer Yellow (50 µM) was used as internal control (*P*_*app*_ < 15·10^−6^ cm/s). LY fluorescence was measured in a 96-well plate with a Fluoroskan Ascent Microplate Fluorometer (Thermo Fisher Scientific). Compounds were dissolved in Ringer-HEPES at a concentration of 200 µM. Then, 500 µL of the compound and 1,500 µL of Ringer-HEPES alone were introduced in the apical or in the basolateral compartments, respectively. The plates were set on at 37 °C for 2 h. The solutions from both compartments were then recovered and quantified by HPLC and identified by MALDI-TOF.

### Peptide Stability in Human Serum

Peptides were dissolved in 1 mL of HBSS buffer/human serum (from human male AB plasma; Sigma-Aldrich) 10:90 (v/v) at a final concentration of 200 µM and incubated at 37 °C for 24 h. Aliquots of 50 µL were extracted at a range of incubation times and treated with 200 µL of cold CH_3_OH (4 °C) to precipitate serum proteins. After 30 min, samples were centrifuged at 13,000 g and 4 °C for 30 min. Supernatants were analyzed by RP-HPLC-PDA and MALDI-TOF MS, using similar procedures as for the quantification of the *in vitro* cell-based BBB model.

### Statistical Analysis

The t-test was for the comparison of two groups. A one-way ANOVA test was selected for multiple comparison analyses. A 5% level was chosen for significance of group. Prior to the statistical data analyses, raw data was sublimated to the Grubb’s test for the detection of outliers.

## Electronic supplementary material

Supplementary information

## Data Availability

All the relevant data supporting the findings are available from the corresponding author on reasonable request.
