# Supplementary material for: HAI Peptide and Backbone Analogs—Validation and Enhancement of Biostability and Bioactivity of BBB Shuttles
Source: Sci Rep. 2018 Dec 18;8:17932. doi: 10.1038/s41598-018-35938-8 (PMC6298966; doi:10.1038/s41598-018-35938-8)
Supplement: Supplementary file 1 — Supplementary information [file 41598_2018_35938_MOESM1_ESM.pdf]

SUPPORTING INFORMATION

HAI Peptide and Backbone Analogs—Validation and Enhancement of Biostability and Bioactivity of BBB Shuttles

Pol Arranz-Gibert,[a#], Roger Prades,[a#] Bernat Guixer,[a] Simón Guerrero,[b,c] Eyleen Araya,[c,d] Sonia Ciudad,[a] Marcelo J. Kogan,[b,c] Ernest Giralt,[a,e]* Meritxell Teixidó[a]*

Affiliations:

a) Institute for Research in Biomedicine (IRB Barcelona), Barcelona Institute of Science and Technology (BIST), Baldiri Reixac 10, Barcelona, E-08028, Spain.

b) Department of Pharmacological and Toxicological Chemistry, Faculty of Pharmaceutical Sciences, University of Chile, Sergio Livingstone 1007, Independencia, Santiago, Chile

c) Advanced Center for Chronic Diseases (ACCDiS), Sergio Livingstone 1007, Independencia, Santiago, Chile

d) Departamento de Ciencias Químicas, Facultad de Ciencias Exactas, Universidad Andres Bello, Av. Republica 275, Santiago, Chile.

e) Department of Inorganic and Organic Chemistry, University of Barcelona, Martí i Franquès 1-11, Barcelona, E-08028, Spain.

# These authors contributed equally.

Corresponding authors (*):

Dr. Meritxell Teixidó and Prof. Ernest Giralt, Institute for Research in Biomedicine (IRB Barcelona), Baldiri Reixac 10, Barcelona, E-08028, Spain. Tel.: +34 93 4037125, Fax: +34 93 4037126.

E-mail: ernest.giralt@irbbarcelona.org, meritxell.teixido@irbbarcelona.org

Table of Contents

Characterization of the Peptides S3

MTT Toxicity Assay S5

Characterization of the AuNPs S6

Peptide Stability in Human Serum S10

In vitro Bovine BBB Cell-Based Model Assay S11

In vitro Human BBB Cell-Based Model Assay S12

Abbreviations S13

References S16

Characterization of the Peptides

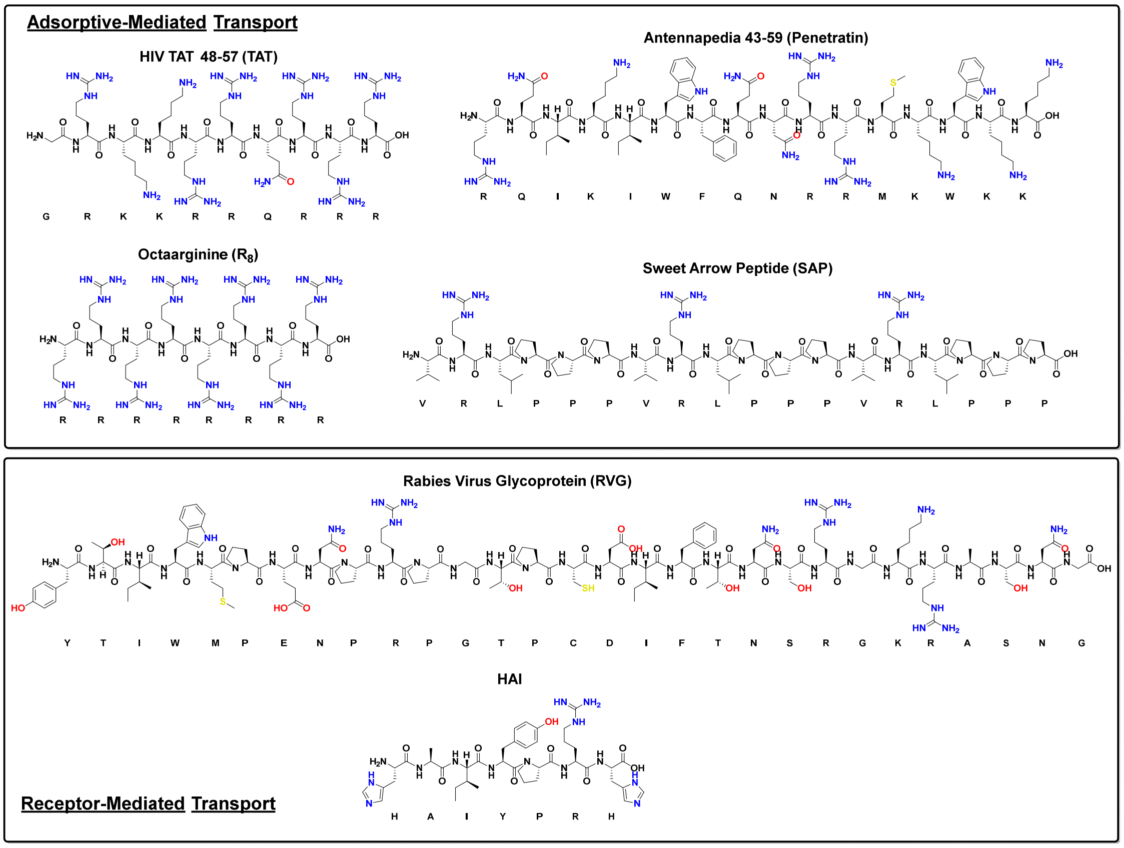

Figure S1. – BBB shuttle candidates classified on the basis of the internalization pathway used, namely adsorptive- or receptor-mediated transport. Functional groups of side-chains are colored per heteroatom type.

Characterization of the Initial Candidate Peptides, (*N*Me)HAI and *r*D-HAI. Total yields: **TAT**, 7.3%; **Cf-TAT**, 8.2%; **antennapedia**, 14.7%; **Cf-antennapedia**, 9.1%; **R$_8$**, 2.1%; **Cf-R$_8$**, 1.8%; **SAP**, 21.1%; **Cf-SAP**, 14.5%; **RVG**, 7.0%; **Cf-RVG**, 9.3%; **HAI**, 19.9%; **Cf-HAI**, 18.2%; ***r*D-HAI**, 8.5%; **Cf-*r*D-HAI**, 10.3%; **(*N*Me)HAI**, 1.1%; **Cf-(*N*Me)HAI**, 2.6%.

**Peptide ID**	**MW (g/mol)**	**HPLC t_R (min)**	**Purity[a] (%)**	**MALDI-TOF $[M+H]^+$**
H-GRKKRRQRRR-OH (**TAT**)	1396.6	3.1	98	1397.0
Cf-GRKKRRQRRR-OH (**Cf-TAT**)	1755.0	3.7	96	1754.8
H-RQIKIWFQNRRMKWKK-OH (**antennapedia**)	2246.7	6.2	95	2246.7
Cf-RQIKIWFQNRRMKWKK-OH (**Cf-antennapedia**)	2605.0	5.6	95	2604.5
H-RRRRRRRR-OH (**R$_8$**)	1267.5	1.2	96	1267.7
Cf-RRRRRRRR-OH (**Cf-R$_8$**)	1625.8	3.7	97	1625.7

H-(VLRPPP)3-OH (**SAP**)	1997.5	4.3	98	1997.2
Cf-(VLRPPP)3-OH (**Cf-SAP**)	2355.8	4.9	99	2356.5
H-YTIWMPENPRPGTPCDIFTNSRGKRASNG-OH (**RVG**)	3266.6	4.1	98	3265.9
Cf-YTIWMPENPRPGTPCDIFTNSRGKRASNG-OH (**Cf-RVG**)	3625.0	4.5	>99	3625.1
H-HAIYPRH-NH_2 (**HAI**)	892.0	4.4	97	892.4
Cf-HAIYPRH-NH_2 (**Cf-HAI**)	1250.3	4.5	99	1251.2
H-hrpyiah-NH_2 (***r*D-HAI**)	892.0	3.1	>99	892.5
Cf-hrpyiah-NH_2 (**Cf-*r*D-HAI**)	1251.3	4.1	99	1251.6
H-HA(NMe)I(NMe)YPR(NMe)H-NH_2 (**(*N*Me)HAI**)	934.0	3.2	97	934.6
Cf-HA(NMe)I(NMe)YPR(NMe)H-NH_2 (**Cf-(*N*Me)HAI**)	1292.3	4.2	96	1292.6

[a]After purification by RP-HPLC.

Table S1. Characterization of the initial candidates, (*N*Me)- and *r*D-peptides studied by RP-HPLC at 220 nm (gradient from 0 to 100% CH_3CN in 8 min; SunFire C_{18} column) and by MALDI-TOF.

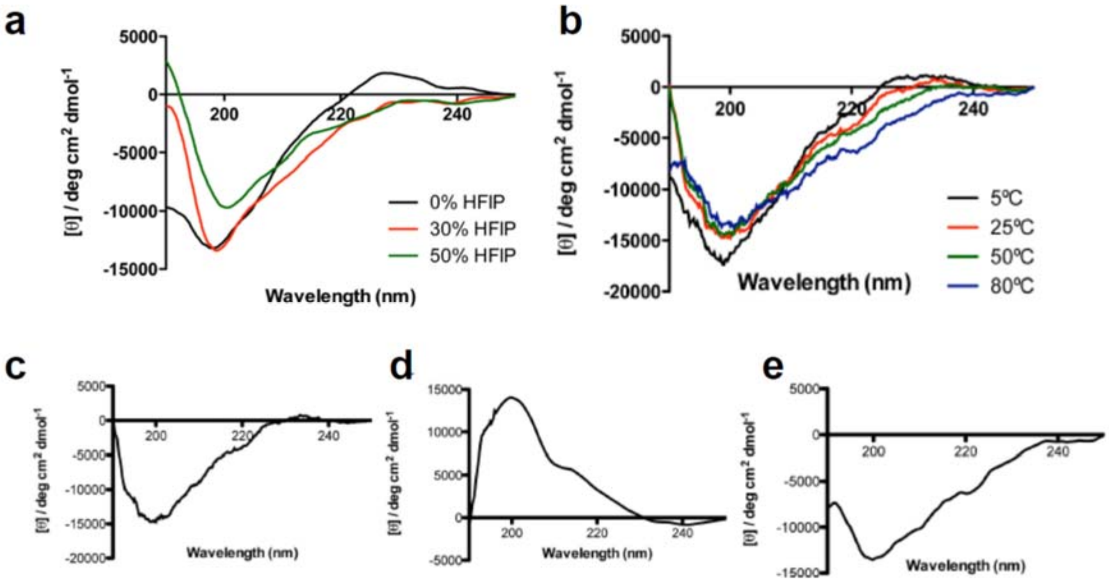

Figure S2. Circular dichroism (CD) traces for (**a**, **b**, **c**) HAI in several conditions, (**d**) *r*D-HAI and (**e**) (*N*Me)HAI at 50 μM, 10 mM phosphate buffer (pH 7.4) and room temperature (except in **b**). In (**a**) and (**b**) several conditions of concentration of HFIP (0, 30 and 50%) or diverse temperatures (5, 25, 50, 80ºC) are used during the recording of the CD spectra, respectively.

MTT Toxicity Assay

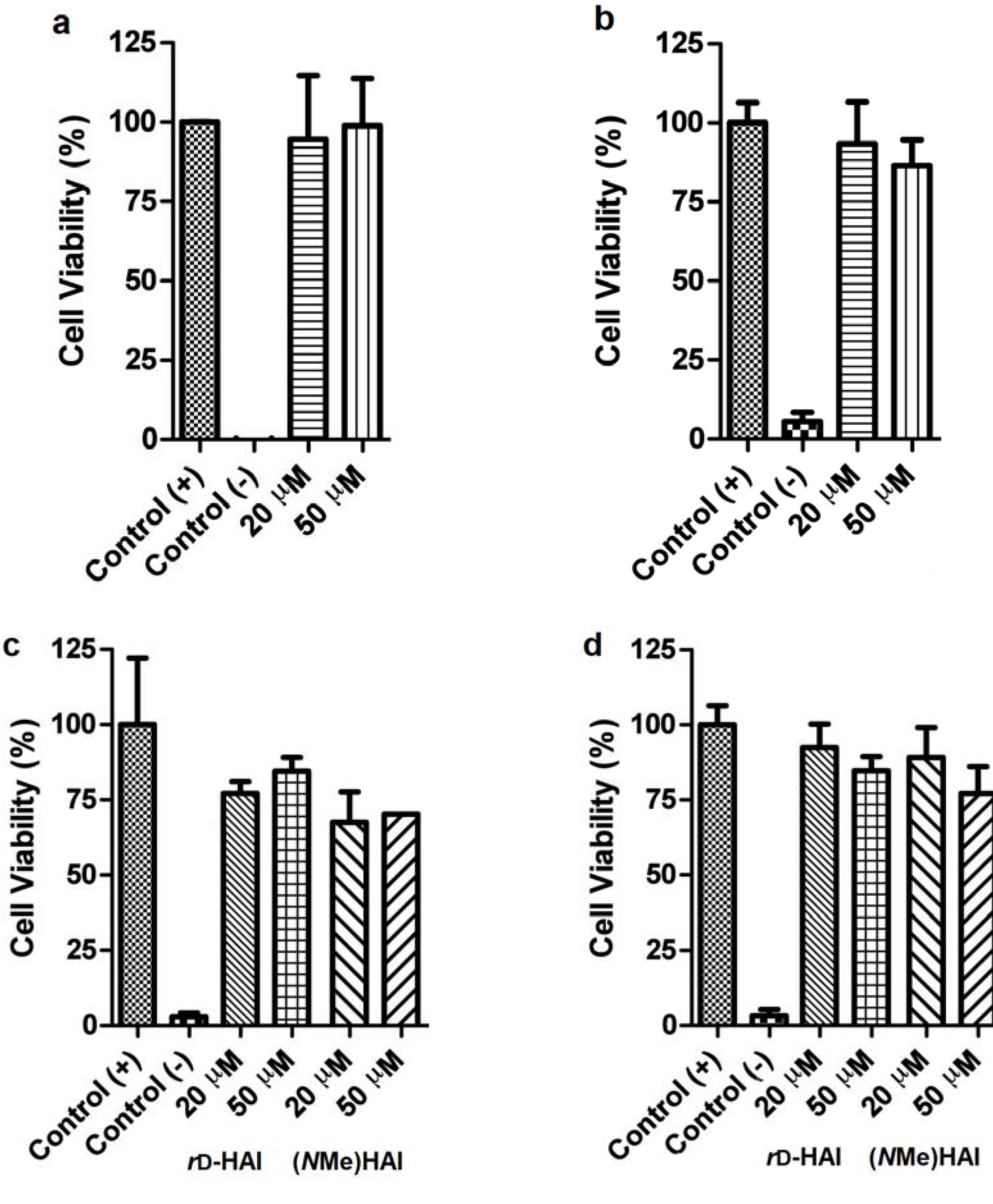

Figure S3. MTT assay using (**a**, **c**) BBECs or (**b**, **d**) rat astrocytes. Cell viability results for peptides (**a**, **b**) **HAI**, (**c**) **(*N*Me)HAI** and (**d**) ***r*D-HAI** at two concentrations (20 and 50 μM). Data are shown as mean ± SD.

Characterization of the AuNPs

A colloidal solution of AuNP was synthesized in the presence of sodium citrate to obtain a solution with a uniform particle size distribution. The AuNP was modified with THR or HAI by mixing AuNP and a peptide stock solution (THR conjugates data, extracted from Prades *et al.*)[1]. UV monitoring showed that the conjugation of the peptide to the AuNP induced a red shift in the surface plasmon resonance band of the colloidal AuNP from 519 to 529 nm when THR or HAI was attached (**Figure S4**). These conjugates were also characterized by TEM observing the presence of a peptide layer that capped the NPs (**Figure S5**).

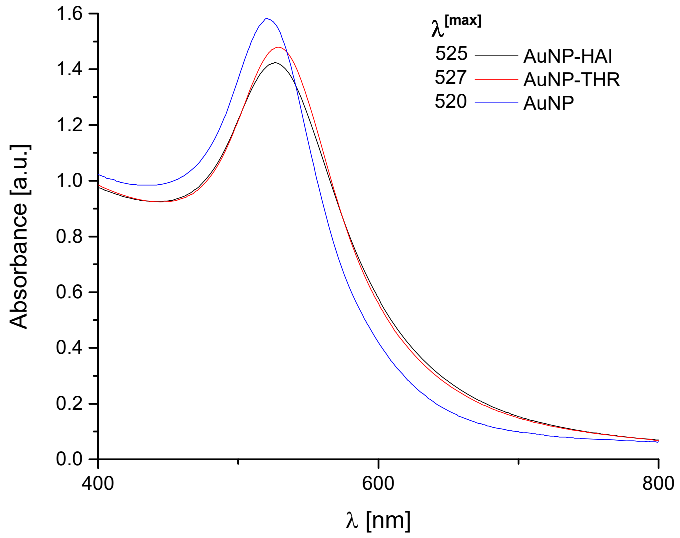

Figure S4. UV-Vis Spectra of AuNP, AuNP-THR and AuNP-HAI.

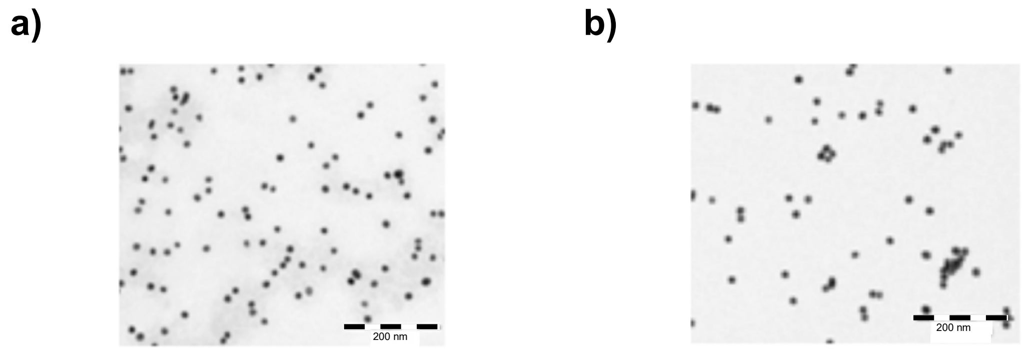

Figure S5. TEM micrograph of (**a**) AuNP-HAI and (**b**) AuNP-THR.

To estimate the number of THR or HAI peptide copies per AuNP, two aliquots of the conjugate were taken to perform two separate determinations. One of the aliquots was used to perform AAA. For this purpose, the sample was centrifuged and the pellet was hydrolyzed with HCl. Later, it was derivatized and the amino acid concentration of the sample was determined by HPLC. In the second aliquot, we determined the concentration of AuNP by ICP-MS taking into account the sizes obtained by TEM. The number of copies per AuNP was calculated by dividing the concentration of peptide by the concentration of NPs. We found 450 ± 160 and 590 ± 200 copies/AuNP, respectively.

In addition, we also performed zeta-potential and dynamic light scattering measurements. The charge and the hydrodynamic diameter of the NPs are crucial parameters to understand their biodistribution and delivery to the brain.

Sample	Z-potential ± deviation (mV)
**AuNP**	-47 ± 5
**AuNP-THR**	-45 ± 7
**AuNP-HAI**	-42 ± 10

Table S2. Zeta-potential of nanoparticles.

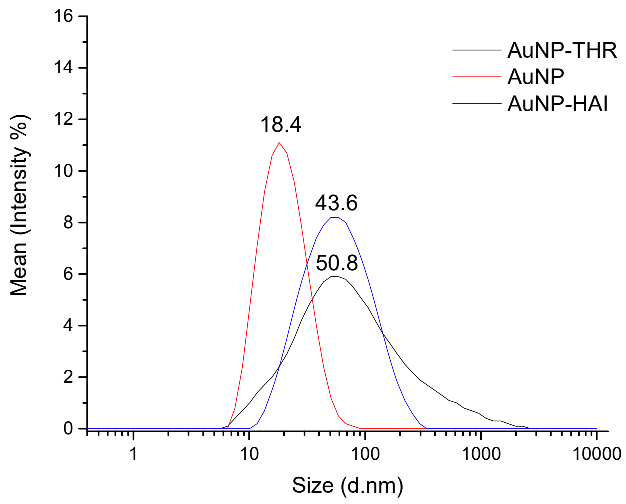

Figure S6. Size distribution of nanoparticles obtained by DLS.

Effects of the AuNP-Peptide Conjugates on Cell Viability. The effects of the peptides and the conjugates AuNP-THR and AuNP-HAI, and AuNP on cell viability was examined using the MTT assay in neuroblastoma SHSY5Y cell line (THR conjugates data, extracted from Prades *et al.*)[1]. These studies allowed us to obtain a profile of the *in vitro* effects of the peptides and AuNP conjugates, crucial data for the validation of our transport studies. No significant effects on cell viability were observed.

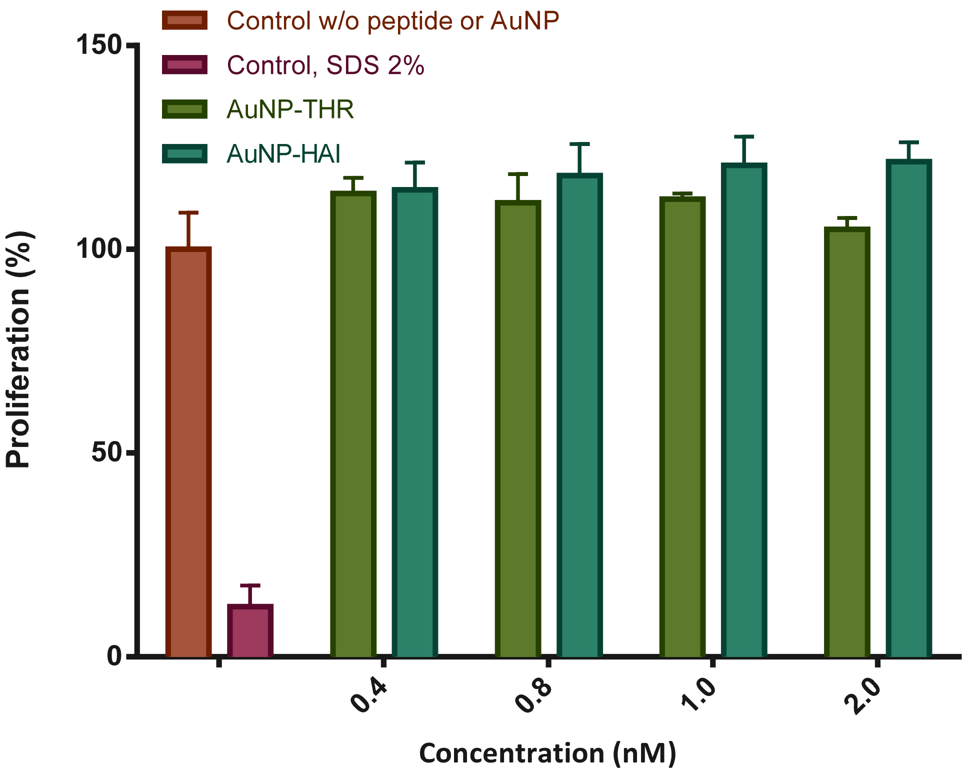

Figure S7. Effect of nanoparticles on cell viability of SH-SY5Y determined by the MTS assay.

***In vitro* Transport of AuNPs in using a Bovine BBB Cell-Based Model Assay**. To assess the capacity of HAI to cross the BBB, this peptide was conjugated to AuNPs and then assayed in an *in vitro* BBB cell-based model. Its transport was compared to the transport displayed by the AuNPs without the peptide. In addition to the transport and apparent permeability, the membrane retention was determined.

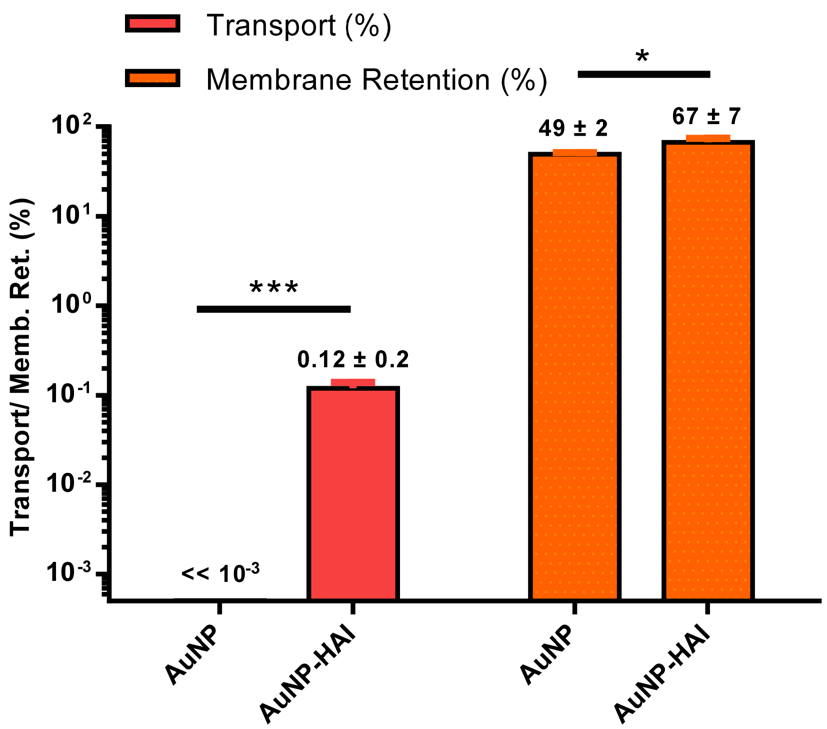

Figure S8. – *In vitro* transport of AuNPs using the same *in vitro* bovine BBB transport model as for the peptides. Membrane retention and transport (logarithmic scale of percentage units) were obtained for both AuNPs and AuNP-HAI. Data are expressed as the mean ± SD.

Peptide Stability in Human Serum

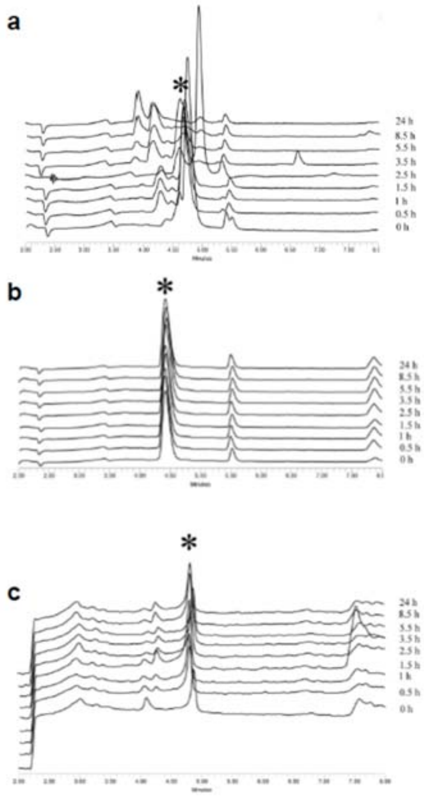

Figure S9. – Stability of (**a**) HAI, (**b**) (*N*Me)HAI and (**c**) *r*D-HAI in human serum: overlaid RP-HPLC traces. HPLC were recorded using a 0-50% B gradient in 8 min (A = 0.045% TFA in H_2O, and B = 0.036% TFA in CH_3CN). Asterisk (*) denotes the peak corresponding to the non-metabolized peptide.

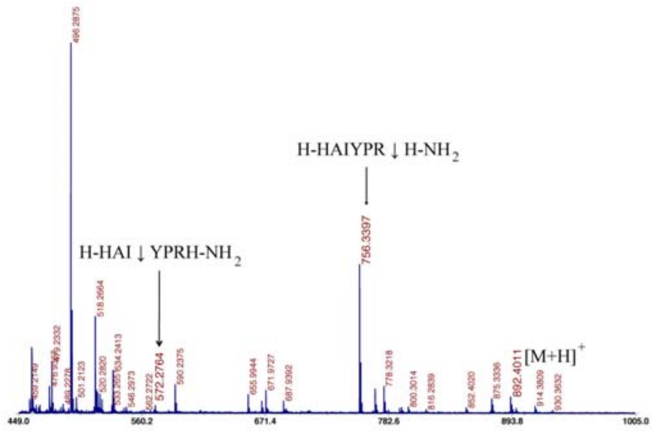

Figure S10. – Stability of HAI in human serum: MALDI-TOF trace at 60 min.

In vitro Bovine BBB Cell-Based Model Assay

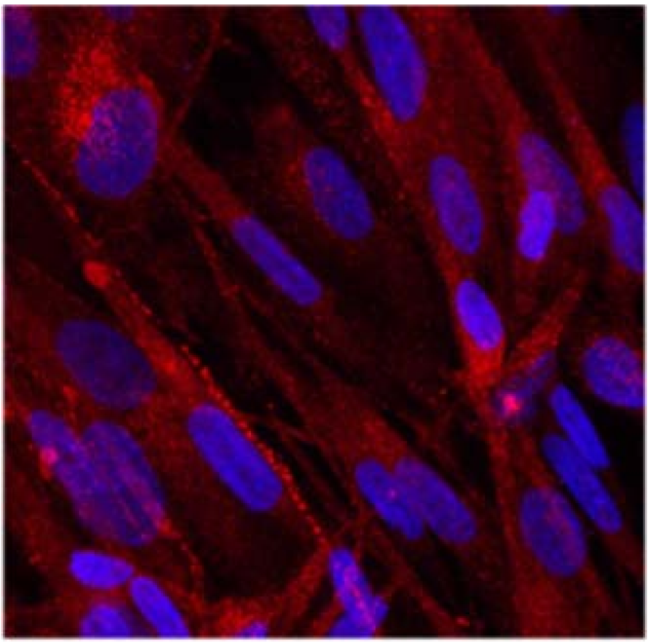

Figure S11. Expression of TfR of BBECs growing in co-culture conditions. Immunochemistry of TfR shown in red, and nuclei stained in blue.

***In vitro* Human BBB Cell-Based Model Assay**

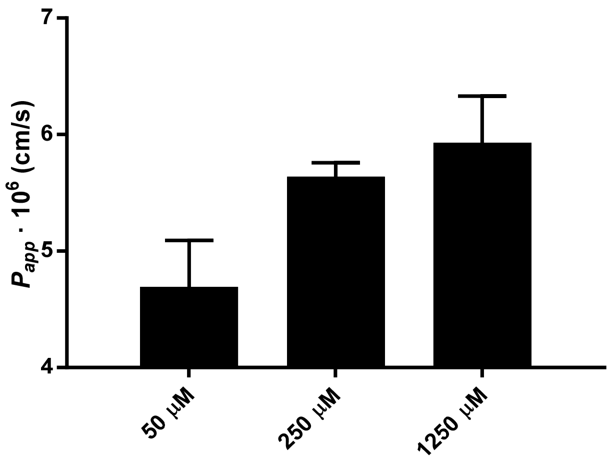

Figure S12. Peptide transport results (mean ± SD; n = 3) using the *in vitro* human BBB cell-based model assay. Peptide *r*D-HAI was assayed at three concentrations (50, 250 and 1250 µM).

Abbreviations

AAA	amino acid analysis
AMT	adsorptive-mediated transcytosis
AuNPs	gold nanoparticles
BBB	blood-brain barrier
BBECs	bovine brain endothelial cells
BSA	bovine serum albumin
Caco-2	human colorectal adenocarcinoma cell line
CD	circular dichroism
Cf	5(6)-carboxyfluorescein
CLSM	confocal laser scanning microscopy
CNS	central nervous system
CPP	cell-penetrating peptide
Cys	cysteine
DBU	1,8-diazabicyclo[5.4.0]undec-7-ene
DCM	dichloromethane
DIEA	*N,N*-diisopropylethylamine
DMEM	Dulbecco's modified Eagle medium
DMF	dimethylformamide
EDT	1,2-ethanedithiol
ESI	electrospray ionization mass spectrometry
Fmoc	9-fluorenylmethoxycarbonyl
HBSS	Hanks' balanced salt solution
HeNe	helium–neon
HEPES	4-(2-hydroxyethyl)-1-piperazineethanesulfonic acid
HFIP	hexafluoro-2-propanol
HIV-1	human immunodeficiency virus 1
HOBt	1-hydroxybenzotriazole
HRMS	high-resolution mass spectrometry

ICP-MS	inductively coupled plasma mass spectrometry
i.p.	intraperitoneal
LY	lucifer yellow lithium salt
MALDI-TOF	matrix-assisted laser desorption/ionization time-of-flight
MS	mass spectrometry
MTBE	methyl *tert*-butyl ether
MTT	3-(4,5-dimethylthiazol-2-yl)-2,5-diphenyltetrazolium bromide
nAChR	nicotinic acetylcholine receptor
NADH	nicotinamide adenine dinucleotide
o-NBS	*o*-nitrobenzensulfonyl chloride
p-MBHA	*p*-methylbenzhydrylamine
P_{app}	apparent permeability
PDA	photodiode array
PyBOP	benzotriazol-1-yl-oxytripyrrolidinophosphonium hexafluorophosphate
QDs	quantum dots
R_8	octaarginine
retro-D-version	peptide made of D-amino acids and with the inverted sequence of a parent peptide
RMT	receptor-mediated transcytosis
RP-HPLC	reversed-phase high-performance liquid chromatography
RVG	rabies virus glycoprotein
SAP	sweet arrow peptide
SD	standard deviation
SDS	sodium dodecyl sulfate
SHSY5Y	thrice-cloned sub-line of SK-N-SH human cell line, isolated from a bone marrow biopsy taken from a four-year-old female with neuroblastoma
siRNA	small interfering RNA
SPPS	solid-phase peptide synthesis
T	transport

TBTU	2-(1*H*-benzotriazol-1-yl)-1,1,3,3-tetramethyluronium tetrafluoroborate
*t*Bu	*tert*-butyl
TEER	transendothelial electrical resistance
TEM	transmission electron microscopy
TFA	trifluoroacetic acid
Tf	transferrin
TfR	transferrin receptor
TIS	triisopropylsilane
t_R	retention time
UV/Vis	ultraviolet/visible

References

1 Prades, R. *et al.* Delivery of gold nanoparticles to the brain by conjugation with a peptide that recognizes the transferrin receptor. *Biomaterials* **33**, 7194-7205, (2012).
